# Spatio-temporal distribution and hotspots of *Plasmodium knowlesi* infections in Sarawak, Malaysian Borneo

**DOI:** 10.1038/s41598-022-21439-2

**Published:** 2022-10-14

**Authors:** Nur Emyliana Yunos, Hamidi Mohamad Sharkawi, King Ching Hii, Ting Huey Hu, Dayang Shuaisah Awang Mohamad, Nawal Rosli, Tarmiji Masron, Balbir Singh, Paul Cliff Simon Divis

**Affiliations:** 1 Malaria Research Centre, Faculty of Medicine and Health Sciences, Universiti Malaysia Sarawak, Kota Samarahan, Sarawak Malaysia; 2 Ministry of Health Malaysia, Kapit Divisional Health Office, Kapit, Sarawak Malaysia; 3 Ministry of Health Malaysia, Kapit Hospital, Kapit, Sarawak Malaysia; 4 Centre for Spatially Integrated Digital Humanities, Faculty of Social Sciences and Humanities, Universiti Malaysia Sarawak, Kota Samarahan, Sarawak Malaysia

**Keywords:** Parasitology, Epidemiology

## Abstract

*Plasmodium knowlesi* infections in Malaysia are a new threat to public health and to the national efforts on malaria elimination. In the Kapit division of Sarawak, Malaysian Borneo, two divergent *P. knowlesi* subpopulations (termed Cluster 1 and Cluster 2) infect humans and are associated with long-tailed macaque and pig-tailed macaque hosts, respectively. It has been suggested that forest-associated activities and environmental modifications trigger the increasing number of knowlesi malaria cases. Since there is a steady increase of *P. knowlesi* infections over the past decades in Sarawak, particularly in the Kapit division, we aimed to identify hotspots of knowlesi malaria cases and their association with forest activities at a geographical scale using the Geographic Information System (GIS) tool. A total of 1064 *P. knowlesi* infections from 2014 to 2019 in the Kapit and Song districts of the Kapit division were studied. Overall demographic data showed that males and those aged between 18 and 64 years old were the most frequently infected (64%), and 35% of infections involved farming activities. Thirty-nine percent of Cluster 1 infections were mainly related to farming surrounding residential areas while 40% of Cluster 2 infections were associated with activities in the deep forest. Average Nearest Neighbour (ANN) analysis showed that humans infected with both *P. knowlesi* subpopulations exhibited a clustering distribution pattern of infection. The Kernel Density Analysis (KDA) indicated that the hotspot of infections surrounding Kapit and Song towns were classified as high-risk areas for zoonotic malaria transmission. This study provides useful information for staff of the Sarawak State Vector-Borne Disease Control Programme in their efforts to control and prevent zoonotic malaria.

## Introduction

Malaria, a mosquito-borne disease, is widely distributed in the tropical and subtropical regions, with more than 400,000 annual deaths reported^1^. Zoonotic malaria by the simian parasite *Plasmodium knowlesi* became prominent since the large focus of cases reported in Kapit division of Sarawak state, Malaysian Borneo almost two decades ago^2^. Knowlesi malaria have been reported in countries across Southeast Asia at low frequency, however, highest prevalence of clinical cases has mainly occurred in Malaysian Borneo^3^. According to the Ministry of Health Malaysia, the prevalence of indigenous malaria caused by human parasites *P. vivax. P. falciparum, P. malariae* and *P. ovale* has shown a remarkable decrease while knowlesi malaria cases have continuously shown an increasing trend with 509 annual cases reported in 2010, to between 1813 and 4124 cases from 2012 to 2020^4^ (Ministry of Health Malaysia, unpublished data). Malaysia is listed by the WHO as one of the countries that has substantially progressed in eliminating malaria by the year 2020^1^. However, zoonotic malaria cases caused by *P. knowlesi* are excluded from the definition of malaria elimination by WHO, which focuses on only the human *Plasmodium* species^1,5,6^.

The malaria-free status by WHO is confirmed if zero incidence of indigenous cases for at least three consecutive years, denoting full interruption of local malaria by *Anopheles* mosquitoes. Nonetheless, certification of malaria-free status for Malaysia by the WHO could be postponed if hundreds cases of knowlesi malaria per year are continuously being reported^7^.

The transmission of *P. knowlesi* has been shown to be complex. To date, there are at least three genetically divergent parasites that can infect humans, based on the analyses of multi-locus microsatellites markers and whole-genome sequences derived from clinical samples across Malaysia^8–11^. Two divergent subpopulations of *P. knowlesi* have been identified sympatrically in human infections in the Kapit division^8,9,12^; one (termed Cluster 1 subpopulation) is associated with long-tailed macaques (*Macaca fascicularis*) and the other (Cluster 2) is linked to pig-tailed macaques (*Macaca nemestrina*). Additionally, an exclusive *P. knowlesi* subpopulation (Cluster 3) has also been described in Peninsular Malaysia, indicating allopatric divergence from Cluster 1 and 2 subpopulations of the Malaysian Borneo due to geographic separation by the South China Sea^10^.

Further large-scale genotyping surveillance using simple PCR tools showed that the *P. knowlesi* Cluster 1 subpopulation is consistently predominant across Malaysian Borneo, and in the Kapit division of Sarawak, Malaysian Borneo accounts for two thirds of all cases with no significant temporal changes over the past 18 years^13^.

It has been suggested that man-made activities and environmental modifications trigger the increasing cases of knowlesi malaria^4,14^. Human activities at the forest or forest fringe are one of the main risk factors for *P. knowlesi* infections, as it requires the presence of both macaque hosts and forest-dwelling *Anopheles* mosquitoes^15,16^. Individuals engaged in agricultural activities, hunting, and logging contribute to almost all cases of *P. knowlesi* infections in both Sarawak and Sabah states of Malaysian Borneo^4,15,17^. With the increasing cases and existence of two sympatric subpopulations in Malaysian Borneo, it is important to determine the spatio-temporal patterns of these *P. knowlesi* subpopulations particularly in the Kapit division of Sarawak where high incidence occurs, as well as the association with environmental changes in order to understand the transmission dynamics of this zoonotic parasite. Therefore, this study aimed to identify hotspots of *P. knowlesi* infections in the Kapit division and to determine the association with risk activities at a geographical scale.

## Methods

### Study area and blood sampling

**Figure 1:**
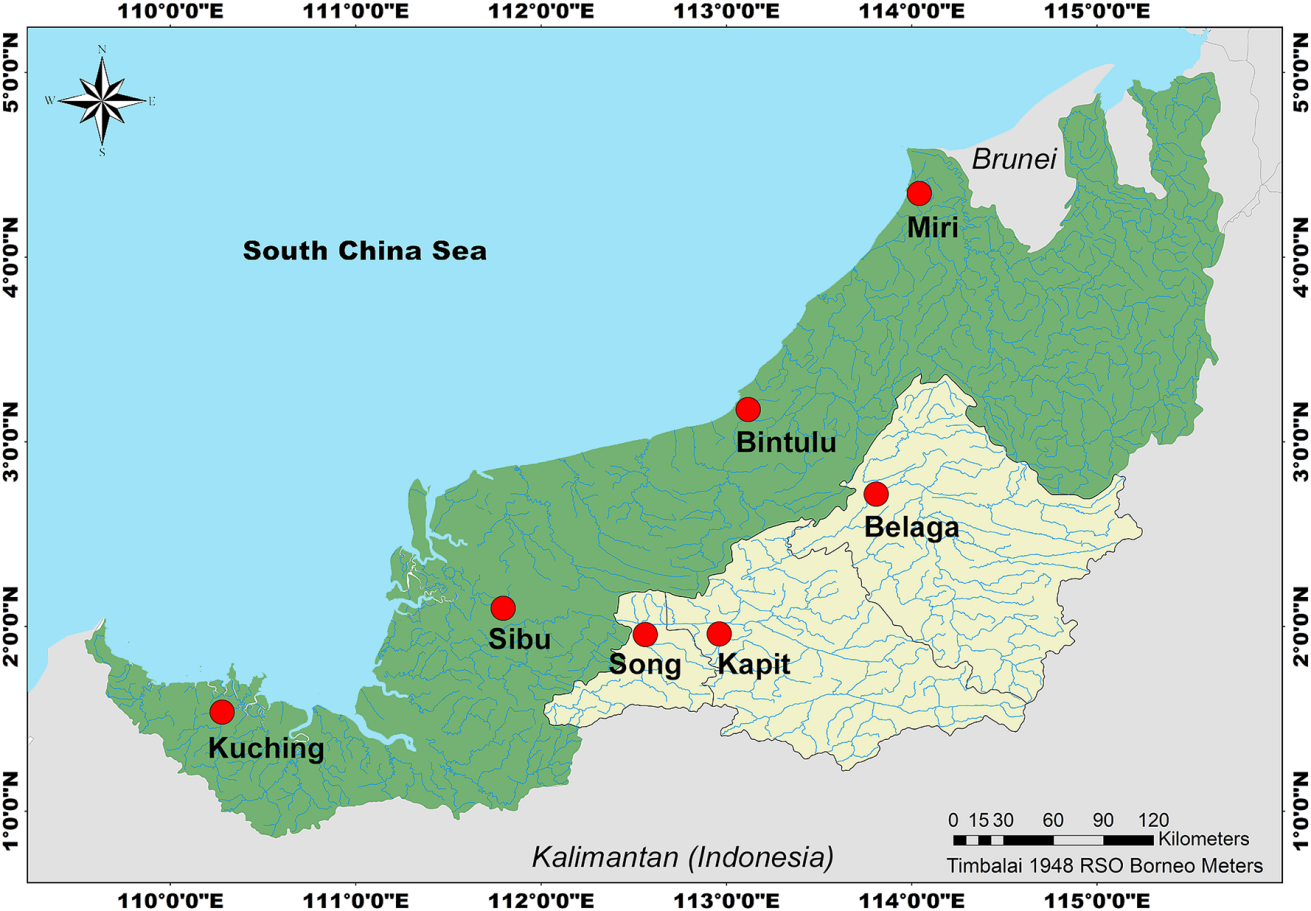
Map of Sarawak state, Malaysian Borneo, showing major rivers and tributaries. Kapit division (in yellow) is located at the central region of Sarawak, bordering Kalimantan of Indonesia. Major cities/towns are shown in red dots. Map was constructed using ArcMap® software v10.3 by Esri (www.esri.com).

This study was conducted in the Kapit division, Sarawak, located at the central part of Malaysian Borneo (Fig. 1). Kapit division consists of three districts covering an area of 38,934 km^2^ with 134,000 inhabitants, mostly (49%) residing in Kapit, followed by Belaga (33%) and Song (18%) districts^18^. According to the Kapit District Council, the topography of the Kapit division varies from lowland to mountainous landscapes, with approximately 80% covered with dense primary forests. The Rejang River and the main upper tributaries, which include Batang Baleh, Batang Katibas, Batang Balui, and Belaga river, flow throughout the division and there were approximately 534 longhouses altogether, most located along the rivers.

Kapit Hospital is the only hospital in the Kapit division, so all patients diagnosed with malaria at government health clinics are referred to this hospital. Approximately 2 mL of venous blood was collected from each patient at Kapit Hospital from June 2018 to December 2019. Each sample was used to prepare blood spots on filter paper and thick and thin blood films, and the remaining blood was frozen. The baseline information, including age, sex, and parasite microscopic examinations were also recorded at the hospital laboratory as part of the routine diagnosis for malaria. All samples were then transferred to the Malaria Research Centre, Universiti Malaysia Sarawak, for further molecular analyses.

Written informed consent was obtained from enrolled patients or parents or guardians for patients below 17 years of age. All procedures were performed in accordance with relevant guidelines outlined in the ethical clearance. Ethical clearance for this study was obtained from the Medical Ethics Committee of Universiti Malaysia Sarawak (UNIMAS/NC-21.02/03-02 Jld.2 (81)), and from the Medical Research and Ethics Committee of the Ministry of Health, Malaysia (NMRR-16-943-31224(IIR)).

### Identification of *Plasmodium* species and genotyping of *Plasmodium knowlesi*

*Plasmodium* DNA was extracted from dried blood spots using InstaGene™ Matrix (Bio-Rad Laboratories, Inc., CA, USA), and the identification of *Plasmodium* species (*P. falciparum, P. vivax, P. ovale, P. malariae*, *P. knowlesi, P. cynomolgi, P. coatneyi,* and *P. inui*) was conducted by nested PCR assays as described previously^2,19^. For infections positive with *P. knowlesi*, each sample was further genotyped using allele-specific PCR assays to identify the two subpopulation clusters^13^.

For a complete 6-year temporal analysis in the current study, we also included a total of 886 genotyping data of *P. knowlesi* infections in the Kapit division from January 2014 to May 2018 obtained from previous studies (Supplementary Fig. S1)^11,13^.

### Demography and geolocation of *P. knowlesi* infections

Demographic data of patients with *P. knowlesi* infections at Kapit division were obtained from the Vector Control Unit, Kapit Divisional Health Office (Ministry of Health Malaysia ethical approval NMRR-17-3210-35624(IIR)). Data of each infection obtained were of a 6-year period from January 2014 to December 2019, which includes gender, ethnicity, occupation, travel history, activities prior to infection, and geographical coordinates. The database was reorganised accordingly in Microsoft Excel by excluding non-*P. knowlesi* infections and other irrelevant information. The map of the Kapit division was obtained from DIVA-GIS free spatial data depository (http://www.diva-gis.org). The coordinate system was projected into Timbalai 1948 RSO Borneo Meters in order to synchronise the digital data structure used in ArcGIS.

Patients were categorised into four age structures based on the dependency ratio in Malaysia^20^, which measures the economic workforce by rationing the number of dependents (non-working age) into the working-age population. Age < 15 and > 64 years old were considered as economically dependent and the age between 15 and 64 was reflected as an active working-age group. In Malaysia, the compulsory schooling age ends at 17 years old, so the working-age was adjusted at 18 years old.

Patients were further categorised into several groups based on their activities two weeks prior to admission to the hospital for malaria. These activities were then categorised according to the distance radius from the longhouses and types of activities. Additionally, occupations were also categorised into six major groups, depending on the distance from the forests and working sites (Supplementary Table S1).

### Distribution pattern analysis

To determine the distribution pattern of *P. knowlesi* infections, the Average Nearest Neighbour (ANN) was used to measure the Nearest Neighbour Ratio (R) based on the observed average distance between the nearest neighbouring infections^21^ (statistical formula for R in the Supplementary Table S2). The distribution pattern of infections is considered clustered when R < 1 while the dispersal pattern is indicated by R > 1^22^. The R-value was validated with Z-scores to test the significance of whether to reject the null hypothesis. The null hypothesis in this study was that there is a random spatial pattern of malaria incidences in the Kapit division.

Kernel density estimation (KDE) interpolation technique was used to determine the hotspot areas of *P. knowlesi* infections using the geolocation data. Kernel density spatial smoothing technique transforms point pattern data into a continuous density map, making it an effective tool to identify hotspot areas of infections^23^. The new thematic layer was created to represent the hotspot of infections in selected time frames.

Due to the disproportion frequencies between Cluster 1 and Cluster 2 *P. knowlesi* subpopulations^13^, normalisation was applied to adjust the default density values of the KDE analysis. This function was set to avoid biased results when determining hotspot areas for the two *P. knowlesi* subpopulations. The density values for each hotspot result were set as default, calculated according to Silverman's Rule of Thumb algorithm in the software programme. To alter the density values, the *P. knowlesi* subpopulation hotspot values were customised according to the total malaria cases density values in that particular year.

## Results

### Prevalence of *Plasmodium* species and *P. knowlesi* subpopulations

We obtained 436 blood samples from malaria patients admitted at Kapit Hospital from June 2018 to December 2019 with parasitaemia ranging from 20 to 384,000 parasites/μl blood (mean parasitaemia 13,221 parasites/μl blood). By PCR assays, 304 (69.7%) were positive for single *P. knowlesi* infections, and 13 (3.0%) had double infections of *P. knowlesi* mixed with other human *Plasmodium* species (Table 1). As expected, we observed more mixed infections by PCR which were not detected by microscopy. Information of the travel history was available for three of the 13 patients with double infections of *P. knowlesi* with other human *Plasmodium* species, and three had recently returned from malaria-endemic countries. The remaining 119 (27.3%) patients identified with non-*P. knowlesi* infections had all recently returned to Kapit after working in timber camps in malaria-endemic countries in Africa, South America and the Western Pacific islands.

**Table 1:** Comparison of the prevalence of *Plasmodium* species between microscopy and nested PCR assays on malaria patients admitted to Kapit Hospital from June 2018 to December 2019.

Infection	*Plasmodium* species by nested PCR	*Plasmodium* species by microscopy					Total by PCR
		Pf	Pk	Pm	Po	Pv	
Single	Pk	4	297	1	0	2	304
	Pf	36	3	1	0	1	41
	Pv	0	6	2	0	53	61
	Pm	0	0	3	0	0	3
	Po	1	0	0	1	3	5
Double	Pk + Pf	1	5	0	0	0	6
	Pk + Pv	0	5	0	0	2	7
	Pf + Pm	1	0	1	0	0	2
	Pf + Po	2	0	0	0	1	3
	Pf + Pv	2	0	0	0	1	3
	Pv + Pm	0	0	0	0	1	1
	Total by microscopy	47	316	8	1	64	436

The species abbreviation corresponds to *Pk*, *P. knowlesi*; *Pf*, *P. falciparum*; *Pm*, *P. malariae*; *Po*, *P.ovale*; *Pv*, *P. vivax.*

Of 317 *P. knowlesi* single or mixed infections, 74.1% (n = 235) were genotyped as Cluster 1 subpopulation and 15.5% (n = 49) were Cluster 2 subpopulation. Mixed genotyped infections were also detected but at low frequency (3.2%, n = 10). There were 23 *P. knowlesi* infections that could not be genotyped using the allele-specific PCR assay, and these were excluded in the subsequent analyses.

Together with *P. knowlesi* infections from previous studies^11,13^, we obtained a total of 1,180 infections throughout the 6-year period from 2014 to 2019, with 68.1% (n = 804) belonging to Cluster 1 subpopulation, 22.8% (n = 269) to Cluster 2 subpopulation and 4% (n = 47) were mixed genotype infections. For subsequent analyses, we excluded 116 infections from the Belaga district since most malaria patients from this district travel to Bintulu hospital instead of Kapit Hospital for treatment. Therefore, a total of 1064 infections from the Kapit and Song districts were analysed (Supplementary Fig. S1).

### Demographic profiles of malaria patients in Kapit and Song districts

From 2014 to 2019, male patients have consistently remained predominant over the years (64%, n = 680, Pearson’s *X*^2^ P < 0.01). Most infections occurred among the active working-age group between 18 and 64 years old (79%, n = 841) with a median age of 41 years old, followed by the elderly above 65 years old (11%, n = 117), school children between 6 and 17 years old (9.6%, n = 102), and only four cases among children below 5 years old.

Occupations of all 1,064 patients were categorised based on the nature of work and distance from the forests (Table 2). A majority (66.4%) of the patients who acquired knowlesi malaria worked near the forests or in the forests, which include short distance working capacity such as farmers, collectors of forest products and fisherman, and long-distance working capacity such as logging camp workers, hunters and road construction workers, respectively. Among the knowlesi malaria patients we also identified housewives and unemployed elderly being the third highest group with 16.4% of the total cases, and a small proportion of school children (1.8%).

**Table 2:** Occupation of *P. knowlesi* patients from Kapit and Song districts admitted in Kapit hospital, 2014 to 2019.

Occupation	2014 (n = 130)	2015 (n = 102)	2016 (n = 160)	2017 (n = 262)	2018 (n = 255)	2019 (n = 155)	Total (n = 1064)
Housewife/elderly	21	16	30	55	33	19	174
Student	13	7	8	15	13	6	62
*Farmer/collecting forest products/fisherman	40	40	52	94	86	43	355
Various workers in towns	16	9	18	17	30	20	110
**Driver/construction worker/logging worker/surveyor/hunter	40	30	50	76	89	67	352
Others (e.g.: oil and gas industry)	0	0	2	5	4	0	11

The term is based on the distance radius from the working site with forest area.

*Defined as short-distance workers.

**Defined as long-distance workers.

We evaluated how the patients acquired *P. knowlesi* infections, and found activities performed within the 2-km radius from the residential areas accounted for most cases (61%, n = 645), and the frequencies were consistent throughout the 6-year period, compared to activities conducted > 2 km from the residential areas (Pearson’s *X*^2^ P = 0.018; Table 3). One third of the patients (n = 352) acquired their infections while farming within a 2-km radius of their longhouses. The next largest group of patients (18.2%, n = 194) were those who acquired their infections while undertaking activities within the longhouse compound, which includes activities near rivers, rearing domestic poultry, and socialising at the common area (*ruai* in local dialect) of the longhouse in the evening. Patients involved in forest logging activities accounted for 14.6% (n = 155) of the total cases, contributing to the highest number of cases conducted more than 2 km away from the residential areas (Table 3).

**Table 3:** Activities performed by *P. knowlesi* patients 2 weeks prior to admission to Kapit Hospital, 2014–2019.

Activities	2014 (n = 130)	2015 (n = 102)	2016 (n = 160)	2017 (n = 262)	2018 (n = 255)	2019 (n = 155)	Total (n = 1064)
**Distance less than 2 km from residential areas**							
Farming	39	36	46	117	73	41	352
Hunting	4	6	11	7	14	15	57
Longhouse activities	24	18	35	48	45	24	194
Activities within school compound	4	5	8	8	10	7	42
**Distance more than 2 km from residential areas**							
Farming > 2 km	11	2	5	1	1	2	22
Hunting > 2 km	5	3	14	10	18	10	60
Labour activities	1	4	4	13	12	10	44
Forest activities	9	2	11	10	14	21	67
Activities at logging camp	8	6	5	17	24	11	71
Logging/timber	25	20	21	31	44	14	155

### Spatio-temporal distribution and hotspots of infections

The overall *P. knowlesi* infections in Kapit and Song districts showed a consistent pattern of clustering distribution from 2014 to 2019 (Nearest Neighbour Ratio, R < 1, P < 0.01, Table 4). The R-value dropped gradually from 2015, indicating a strong clustering pattern that occurred randomly as indicated by the negative Z-score values over the years. Using the Kernel density estimation (KDE) technique, hotspots of infections were identified within a 5-km radius and 20-km radius of Song and Kapit town, respectively (Supplementary Fig. S2). Baleh, an area located 50 km east of Kapit town and has undergone continuous infrastructure development, showed varying hotspot patterns during the 6-year period.

**Table 4:** The Average Nearest Neighbour (ANN) analysis shows the distribution pattern of *P. knowlesi* infections in Kapit and Song districts, 2014–2019.

*P. knowlesi* infection	Year	R	P-value	Z-score	Distribution pattern
Overall (n = 1064)	2014	0.50	< 0.01	− 10.92	Clustered
	2015	0.64	< 0.01	− 6.93	Clustered
	2016	0.46	< 0.01	− 12.93	Clustered
	2017	0.41	< 0.01	− 18.30	Clustered
	2018	0.40	< 0.01	− 18.21	Clustered
	2019	0.39	< 0.01	− 14.52	Clustered
Cluster 1 (n = 732)	2014	0.55	< 0.01	− 7.78	Clustered
	2015	0.72	< 0.01	− 4.45	Clustered
	2016	0.58	< 0.01	− 7.59	Clustered
	2017	0.50	< 0.01	− 12.80	Clustered
	2018	0.44	< 0.01	− 14.49	Clustered
	2019	0.43	< 0.01	− 11.94	Clustered
Cluster 2 (n = 245)	2014	0.70	< 0.01	− 3.80	Clustered
	2015	0.94	0.54	− 0.61	Random
	2016	0.63	< 0.01	− 5.05	Clustered
	2017	0.62	< 0.01	− 5.41	Clustered
	2018	0.66	< 0.01	− 4.36	Clustered
	2019	0.91	0.43	− 0.78	Random

**Figure 2:**
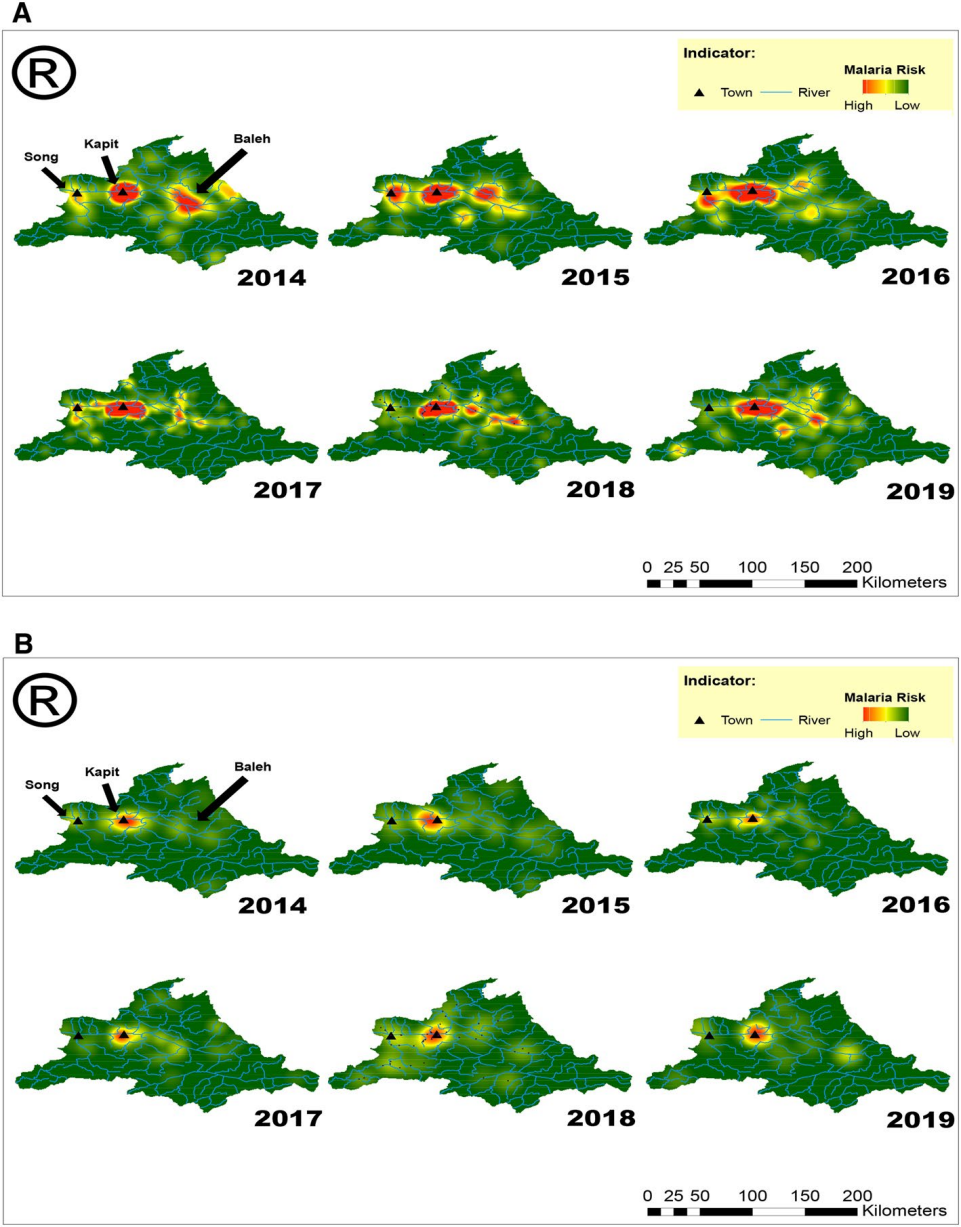
(**A**) Spatio-temporal hotspot analysis of *P. knowlesi* Cluster 1 infections (n = 732) in Kapit and Song districts from 2014 to 2019. The malaria risk is indicated from red as a hotspot (high risk) to green as cool spot (low risk) areas. Maps were constructed using ArcMap® software v10.3 by Esri (www.esri.com). (**B**) Spatio-temporal hotspot analysis of *P. knowlesi* Cluster 2 infections (n = 245) in Kapit and Song districts from 2014 to 2019. The malaria risk is indicated from red as a hotspot (high risk) to green as cool spot (low risk) areas. Maps were constructed using ArcMap® software v10.3 by Esri (www.esri.com).

Similar analyses were also performed independently on patients with *P. knowlesi* Cluster 1 (n = 732) and Cluster 2 (n = 245) infections. Patients with Cluster 1 infection showed similar clustering distribution and hotspots pattern to those of the overall *P. knowlesi* infections (Table 4, Fig. 2A). The Cluster 2 subpopulation, however, showed variation in the patterns throughout the 6-year period. While clustering patterns were observed in most years (R < 1), random distribution patterns were also observed in 2015 (P = 0.54, Z = − 0.61) and 2019 (P = 0.43, Z = − 0.78). Unlike those infected with the Cluster 1 subpopulation, infections of Cluster 2 showed less hotspot areas, with most hotspots confined within the Kapit town vicinity (Fig. 2B).

**Figure 3:**
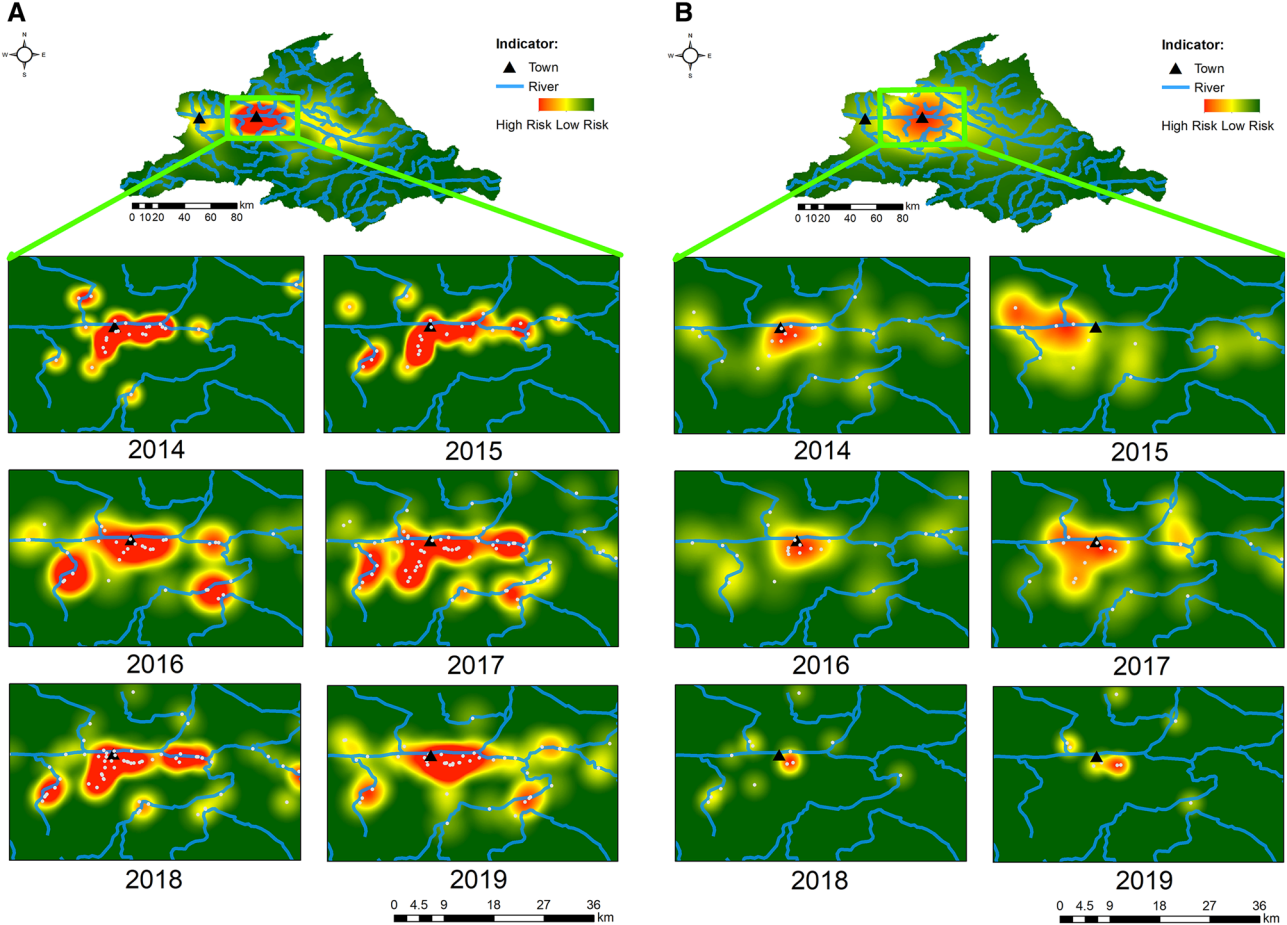
(**A**) Zoom-in of hotspot areas of *P. knowlesi* Cluster 1 infections surrounding Kapit town. The malaria risk is indicated from red as a hotspot (high risk) to green as cool spot (low risk) areas. Maps were constructed using ArcMap® software v10.3 by Esri (www.esri.com). (**B**) Zoom-in of hotspot areas of *P. knowlesi* Cluster 2 subpopulation surrounding the Kapit town. The malaria risk is indicated from red as a hotspot (high risk) to green as cool spot (low risk) areas. Maps were constructed using ArcMap® software v10.3 by Esri (www.esri.com).

**Figure 4:**
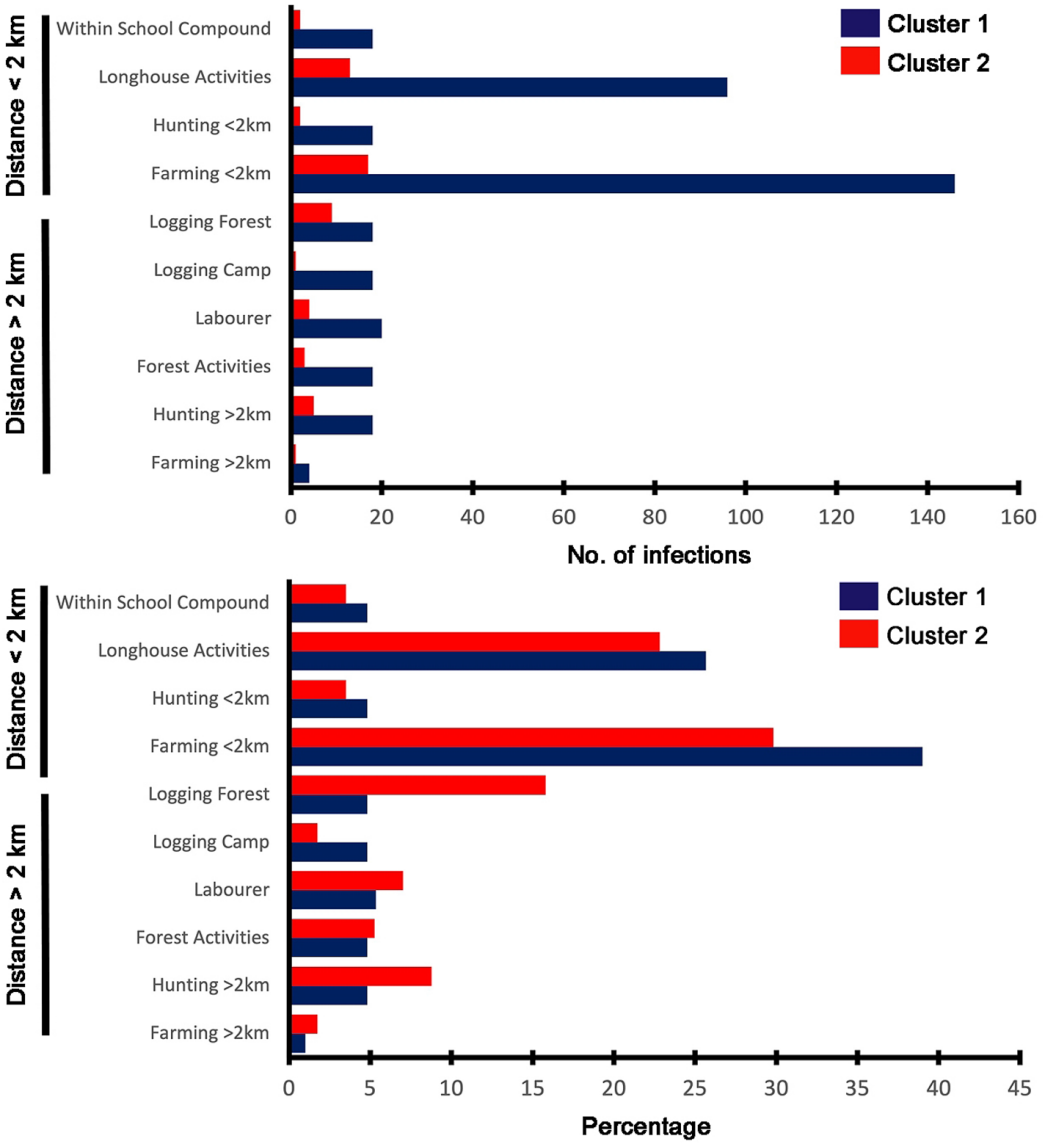
Risk activities among *P. knowlesi* patients with Cluster 1 and Cluster 2 infections within Kapit hotspot zones (refer to Fig. 3). A total of 374 Cluster 1 infections and 57 Cluster 2 infections were identified within this zone.

Since the area surrounding Kapit town has been consistently identified as a hotspot for *P. knowlesi* infections, we reanalysed this area by zooming 13 times for an improved hotspot resolution. Overall, 431 *P. knowlesi* infections were identified within this hotspot zone, with 374 Cluster 1 infections and 57 Cluster 2 infections. In this analysis, the hotspot zones were well-defined for both subpopulations (Fig. 3), with decreased concentrated hotspot units in 2019. The assessment of risk activities showed a statistically significant difference for both Cluster 1 and Cluster 2 subpopulations (99% CI). Within these hotspot zones, there was an equal ratio between males and females of Cluster 1 infections, and most of these were related to farming < 2 km (39%, n = 146) and activities conducted within the longhouse compound (26%, n = 96) (Fig. 4). In contrast, males were more prevalent for Cluster 2 infections, accounting for two-thirds of the patients.

Activities conducted less than 2 km radius within the longhouses and school compounds contributed more cases compared to activities conducted remotely for both clusters (Fisher’s Exact P = 0.03). For Cluster 1 infected individuals, those engaged in activities near the longhouses or within the school compound acquired knowlesi malaria more than those working at the remote forests (independent *t* (8*)* = − 2.14, P = 0.03). This was not observed for Cluster 2 where both groups of activity type contributed equally to knowlesi malaria infections (independent *t* (8) = − 1.37, P = 0.10).

## Discussion

The forests of Sarawak contribute to the economic development and livelihood support of millions of people in this region, particularly the indigenous communities^24^. Activities that enhance economic growth are mainly related to agricultural expansion, industrial-scale logging, hunting, and infrastructure developments such as hydroelectric dams, and these contribute to the alteration of the forest landscape and loss of biodiversity^25,26^. The encroachment of humans into the forest would expose them to wild macaques and mosquitoes that feed on these animals, resulting in potential zoonoses^16,27^. Males and people aged between 18 and 64 years old were observed to contribute most cases of knowlesi malaria in the current study, and this has also been observed in many previous studies in both Malaysian Borneo and Peninsular Malaysia^3,28,29^. These males were involved in hunting activities, logging and construction work. In contrast, women were mainly homemakers and performed more domestic activities near the longhouses such as subsistence farming.

Consistent with a previous study on the prevalence of two sympatric subpopulations in Malaysian Borneo^13^, Cluster 1 infections have been consistently predominant compared to Cluster 2 infections in the Kapit division, accounting for two-thirds of the total cases observed over the 6-year period, 2014–2019. This is expected since Cluster 1 infection is associated with long-tailed macaques^12^, and this macaque species is known to inhabit areas with close proximity to humans for easy access to food and a result of deforestation^30,31^. Furthermore, these macaques are commonly found in broader ranges of both disturbed and secondary forests in lowland and hilly mountainous areas^32^. In contrast, Cluster 2 infections that are associated with the pig-tailed macaques, accounted for only one-third of the total cases. Compared to long-tailed macaques, these macaques prefer to spend more time in the ground foraging at primary forest and the fringes of oil palm plantations^32,33^. In the East Kalimantan province of Indonesian Borneo, pig-tailed macaques are absent from deforested areas^34^, and it is important to assess whether this applies to the Kapit Division since this information is essential to understand the transmission of this zoonotic disease.

We also observed 23 *P. knowlesi* infections that could not be genotyped into either subpopulation clusters using a genotyping PCR assay that can correctly identify genotypes for patients with a parasitaemia as low as ~ 4 parasites/μl blood^13^. One explanation for this is that there could be variations in the primer binding sites that were not detected when the allele-specific PCR assays were previously designed. Sequencing of the primer binding sites for these 23 samples would reveal whether variations of the DNA sequence are responsible for the failure to genotype.

Despite the different proportions of Cluster 1 and Cluster 2 infections, we observed clustering patterns of distribution of *P. knowlesi* infections, with most infections being acquired near the town areas. The population density is mainly concentrated within the vicinity of 20 km of the Kapit town with better infrastructure developments and amenities. Both hotspot subpopulations have disparity features, where the more medium risk of malaria transmissions was seen in Cluster 2 subpopulations. Progressive insights in the hotspot areas showed that patients with Cluster 2 infections were mostly associated with hunting, logging, working in road and bridge construction and plantations, and those undertaking forest activities, as observed in a previous study from 2016 to 2018 of knowlesi malaria patients at Kapit Hospital^35^. Hence, based on the nature of occupation and forest-related activities, the prevalence was higher in males (71%) compared to females (29%) and occurred remotely in the deep forests for Cluster 2 infections. Cluster 1 infections, however, consisted of an equal ratio of both gender since most of the activities occurred within the radius of longhouses, involving small-scale farming, fishing and poultry rearing.

In recent years, Sarawak has been focusing on reducing the dependence on fossil fuels and non-renewable resources by providing access to sustainable modern energy. Baleh, located approximately 90 km southeast of Kapit town, was selected for the development of a hydroelectric dam in 2015. This resulted in increasing development of infrastructure such as roads, which resulted in forest clearance and altered the habitat of macaques and mosquitoes in the areas^27^. Additionally, construction workers were exposed to mosquitoes that feed on wild macaques during forest clearance, especially pig-tailed macaques, increasing the chances in acquiring Cluster 2 *P. knowlesi* infections. Due to this, the hotspot of knowlesi malaria in this area may be related to the development of the Baleh area because of forest clearance. A similar observation was made in the neighbouring state of Sabah, where predictive analysis showed there was a strong link between deforestation and *P. knowlesi* occurance^27^.

Environmental alteration by humans potentially promotes the presence and abundance of disease vectors^36^. An early molecular entomological study conducted in Kapit district incriminated *Anopheles latens* as the vector, and these mosquitoes are predominantly found at forest fringes in farming areas^37^. Recent entomological surveillance conducted at low zoonotic malaria transmission areas in Betong and Lawas districts showed additional potential vectors such *as An. balabacensis, An. donaldi, An. roperi* and *An. collessi*^38,39^. Since both macaque species are widespread and have different behavior, it is unknown whether different mosquitoes feed on these macaques at different habitat types or on selected *P. knowlesi* genotypes. Compared to long-tailed macaques, pig-tailed macaques spend more time in the ground foraging at primary forests and at the fringes of oil palm plantations^33^. Therefore, comprehensive entomology studies to determine the bionomics and the diversity of *Anopheles* mosquito species, genotyping of divergent *P. knowlesi* parasites, combined with studies to map the distribution of the two macaque species are essential to understand the transmission of both genetically divergent *P. knowlesi* subpopulations.

The available evidence strongly suggests that knowlesi malaria is a zoonosis and that the primary hosts are long-tailed and pig-tailed macaques^13^. Human-mosquito-human transmission has been demonstrated under experimental conditions in the 1960s^40^ and recent studies have demonstrated the presence of viable gametocytes in knowlesi malaria patients so it is possible and may well be occurring^41,42^. However, proving that such transmission is currently occurring would be extremely difficult since all the cases are occurring in areas close to habitats of the reservoir macaque hosts.

In conclusion, the current study shows that hotspots of *P. knowlesi* infections in the Kapit division are strongly associated with agricultural and forest-related activities. There are significant differences in risk activities between Cluster 1 and Cluster 2 infections, where most cases for Cluster 2 infections are related to deep forest activities. Malaria hotspot analysis provides a useful tool in efforts to control malaria by utilizing time-consistent updates of localities of infection.

## Supplementary Information

Supplementary Information.

## Data Availability

The dataset generated during and/or analysed during the current study are available from the corresponding author on reasonable request.
