# Supplementary material for: Spatio-temporal distribution and hotspots of Plasmodium knowlesi infections in Sarawak, Malaysian Borneo
Source: Sci Rep. 2022 Oct 14;12:17284. doi: 10.1038/s41598-022-21439-2 (PMC9568661; doi:10.1038/s41598-022-21439-2)
Supplement: Supplementary file 1 — Supplementary Information. [file 41598_2022_21439_MOESM1_ESM.docx]

**Supplementary Table S1:** Activities conducted by patients with *P. knowlesi* infections two weeks prior to admission to Kapit Hospital

| Activities | Description |
| --- | --- |
| Farming <2 km* | Farming within a 2 km radius from longhouses |
| Farming >2 km | Farming more than 2 km radius from longhouses |
| Forest activities | Collecting forest product, frog hunting, fishing |
| Hunting <2 km* | Hunting within a 2 km radius from longhouses |
| Hunting >2 km | Hunting more than 2 km radius from longhouses |
| Labour activities | Construction activities on roads, houses, and bridges |
| Activities in logging camp | Any activity that happened within the logging base camp, such as small-scale farming, cooking, and management office |
| Logging | Logging activity in forested areas, such as cutting the tree, heavy vehicles driver, etc |
| Longhouse activities | Swimming at the nearby river, socialising at common areas (ruai) at night, feeding poultry |
| Activities within school compound | Night security guard, night class, football, staying outside of dormitory at night |

*Risk of infection is based on the movement of patients within 2-km radius from their residential areas, which mainly include small-scale farming that requires walking or short drive less than 30 minutes.

**Supplementary Table S2**: Statistical formula for R

| Statistical formula | Description |
| --- | --- |
| $R= \frac{D̅ₒ}{D̅ₑ}$ | - D̅ₒ is the observed mean distance between each infection with the nearest neighbouring individual infection - D̅ₑ is the expected mean distance for the features determined as random pattern |
| $\bar{D}ₒ=\frac{\sum_{i-1}^{n} dᵢ}{n}$ | - dᵢ is the equal distance between each infection and its nearest infection - i is the nearest infection point - n is the total number of infections - A is the total study area |
| $D̅ₑ =\frac{0.5}{\sqrt{n /A}}$ |  |
| $z= \frac{\bar{D}ₒ- D̅ₑ}{SE}$ |  |
| $SE= \frac{0.26136}{\sqrt{n^{2} /A}}$ |  |


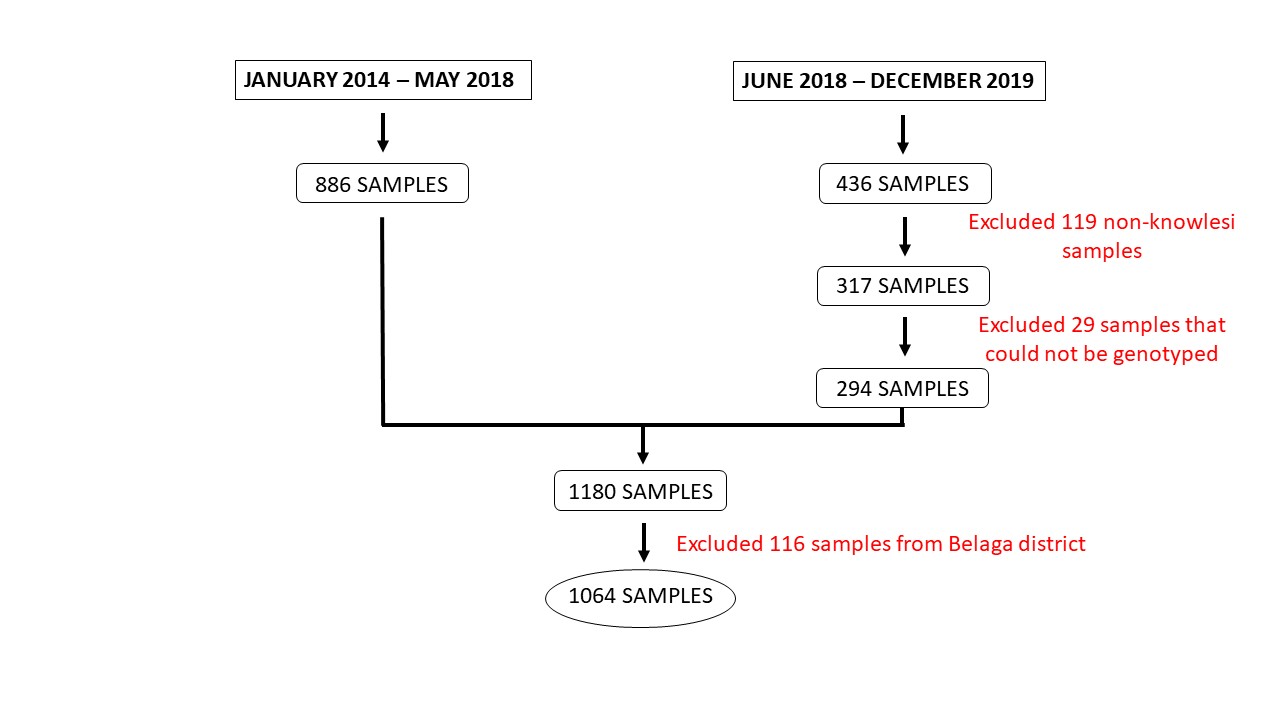


**Supplementary Figure S1**: Flow chart of *Plasmodium knowlesi* sample from 2014 to 2019 for the analyses of spatio-temporal distribution and hotspots of infections

**Supplementary Figure S2:** Spatio-temporal hotspot analysis of *P. knowlesi* infections (n = 1064) in Kapit and Song districts from 2014 to 2019. The malaria risk is indicated from red as hotspot (high risk) to green as cool spot (low risk) areas. Maps were constructed using ArcMap® software v10.3 by Esri (www.esri.com).
